# CD45^dim^CD34^+^CD38^−^CD133^+^ cells have the potential as leukemic stem cells in acute myeloid leukemia

**DOI:** 10.1186/s12885-020-06760-1

**Published:** 2020-04-06

**Authors:** Sook-Kyoung Heo, Eui-Kyu Noh, Lan Jeong Ju, Jun Young Sung, Yoo Kyung Jeong, Jaekyung Cheon, Su Jin Koh, Young Joo Min, Yunsuk Choi, Jae-Cheol Jo

**Affiliations:** 1 Biomedical Research Center, Ulsan University Hospital, University of Ulsan College of Medicine, Ulsan, 44033 Republic of Korea; 2 Department of Hematology and Oncology, Ulsan University Hospital, University of Ulsan College of Medicine, 877 Bangeojinsunhwan-doro, Dong-gu, Ulsan, 44033 Republic of Korea

**Keywords:** Acute myeloid leukemia, Leukemic stem cells, CD45^dim^CD34^+^CD38^−^CD133^+^ cells, Prognosis, Immunophenotyping

## Abstract

**Background:**

Leukemia stem cells (LSCs) in play an important role in the initiation, relapse, and progression of acute myeloid leukemia (AML), and in the development of chemotherapeutic drug resistance in AML. Studies regarding the detection of LSCs and the development of novel therapies for targeting them are extensive. The identification of LSCs and targeting therapies for them has been continuously under investigation.

**Methods:**

We examined the levels of CD45^dim^CD34^+^CD38^−^CD133^+^ cells in bone marrow samples from patients with hematological malignancies and healthy controls, using four-color flow cytometry.

**Results:**

Interestingly, the CD45^dim^CD34^+^CD38^−^CD133^+^ cells were highly expressed in the bone marrow of patients with AML compared to that in healthy controls (HC). Moreover, the proportions of CD45^dim^CD34^+^CD38^−^CD133^+^ cells were also examined in diverse hematological malignancies, including AML, CML, DLBCL, MM, MDS, HL, ALL, and CLL. LSCs were prominently detected in the BMCs isolated from patients with AML and CML, but rarely in BMCs isolated from patients with DLBCL, MM, MDS, ALL, CLL, and HL. Additionally, the high CD45^dim^CD34^+^CD38^−^CD133^+^ cell counts in AML patients served as a significantly poor risk factor for overall and event free survival.

**Conclusions:**

Therefore, our results suggest that CD45^dim^CD34^+^CD38^−^CD133^+^ cells in AML might potentially serve as LSCs. In addition, this cell population might represent a novel therapeutic target in AML.

## Background

Acute myeloid leukemia (AML) is generally regarded as a stem cell disease. It originates from a class of leukemic stem cells that are capable of self-renewal [1, 2]. AML is a heterogeneous disease, with respect to the causative pathogenic mutations and clinical outcomes [3]. AML can progress aggressively within a short period of time and become lethal. Survival rates for adults with AML are very poor despite extensive chemotherapy and/or targeted therapies, provided along with supportive care [4].

The leukemia stem cells (LSCs) in AML play an important role in the development, relapse and progression of leukemia, and in the development of chemotherapeutic drug resistance in AML [5]. Recent studies have suggested that LSCs are capable of giving rise to identical daughter cells that can differentiate into other cells and maintain AML [6, 7]. Rhenen et al. showed that a high percentage of CD34^+^CD38^−^ stem cells at diagnosis significantly correlated with a high minimal residual disease frequency and subsequently to relapse in AML patients. These cell populations directly correlated with poor survival [8, 9].

Identification and characterization of the LSC population is one of the best ways to develop treatment strategies and to improve treatment outcomes in patients with AML and other malignant diseases [3]. Extensive basic research on the identification and targeting of LSCs is being done globally. Many scientists are interested in this area and have found appropriate biological markers for LSC population in AML, including CD34^+^CD38^−^ cells [10, 11], CD34^+^lin^−^ cells [12], CD34^+^Thy1^+^CD38^low^ cells [13], CD34^+^CD117^+^ cells [14], CD34^+^CD38^−^CD123^+^ cells [15–17], CD34^+^CD38^−^CD123^+^CD33^+^ cells [18], CD34^+^CD38^−^C-type lectin-like molecule-1 (CLL-1)^+^ cells [19], CD34^+^CD38^−^CD96^+^ cells [20], CD34^+^CD38^−^CD45^−/low^ cells [21], CD34/CD123/CD25/CD99^+^ [5], etc. The CD34^+^CD38^−^ progenitor cells express varying levels of the target receptors, CD33, CD133, and c-kit (CD117) [22]. However, most of the studies have not provided any conclusive data. Therefore, we analyzed the findings from published studies and identified new combinations that could help detect LSCs. We developed this method using the basic CD34^+^CD38^−^ markers to which novel antigens such as CD45^dim^ and CD133 were added. In this study, we developed a four-color flow cytometric analysis method, and measured the levels of LSCs in bone marrow cells isolated from AML patients. Our findings suggest that CD45^dim^CD34^+^CD38^−^CD133^+^ cells exhibit similar potential as that of LSCs in AML patients.

## Methods

### Reagents

Mouse anti-human CD45-FITC (Clone 2D1, Cat No. 347463), mouse anti-human CD34-PE [Clone 8G12 (also known as HPCA2), Cat No. 348057], mouse anti-human CD38-PE-Cy™5 (Clone HIT2, Cat No. 555461), and appropriated isotype control antibodies were purchased from BD Biosciences (San Diego, CA, USA). Mouse anti-human CD133-APC (Clone CD133, Cat No. 130–090-826) was obtained from Miltenyi Biotec (San Diego, CA, USA).

### Patient samples

We analyzed bone marrow samples collected from 87 patients who were newly diagnosed with AML (*n* = 40), chronic myeloid leukemia (CML, *n* = 6), diffuse large B-cell lymphoma (DLBCL, *n* = 19), multiple myeloma (MM, *n* = 10), myelodysplastic syndrome (MDS, *n* = 5), Hodgkin lymphoma (HL, n = 4), acute lymphocytic leukemia (ALL, *n* = 3), or chronic lymphocytic leukemia (CLL, *n* = 2). Control bone marrows were obtained to rule out hematologic disorders but proven to be normal marrows from 27 healthy donors at Ulsan University Hospital, Ulsan South Korea. Baseline clinical characteristics of 40 patients with AML are summarized in Supplementary Table 1. Other patient characteristics (expect AML) are summarized in Supplementary Table 2.

### Isolation of bone marrow cells

The bone marrow cells (BMCs) were isolated by the density gradient method, as previously described [23]. In brief, BMCs were isolated via density gradient centrifugation at 400×*g* using Lymphoprep (Axis-Shield, Oslo, Norway; density, 1.077 g/mL). They were washed with phosphate-buffered saline (PBS).

### Flow cytometric phenotypic analysis

**Fig. 1:**
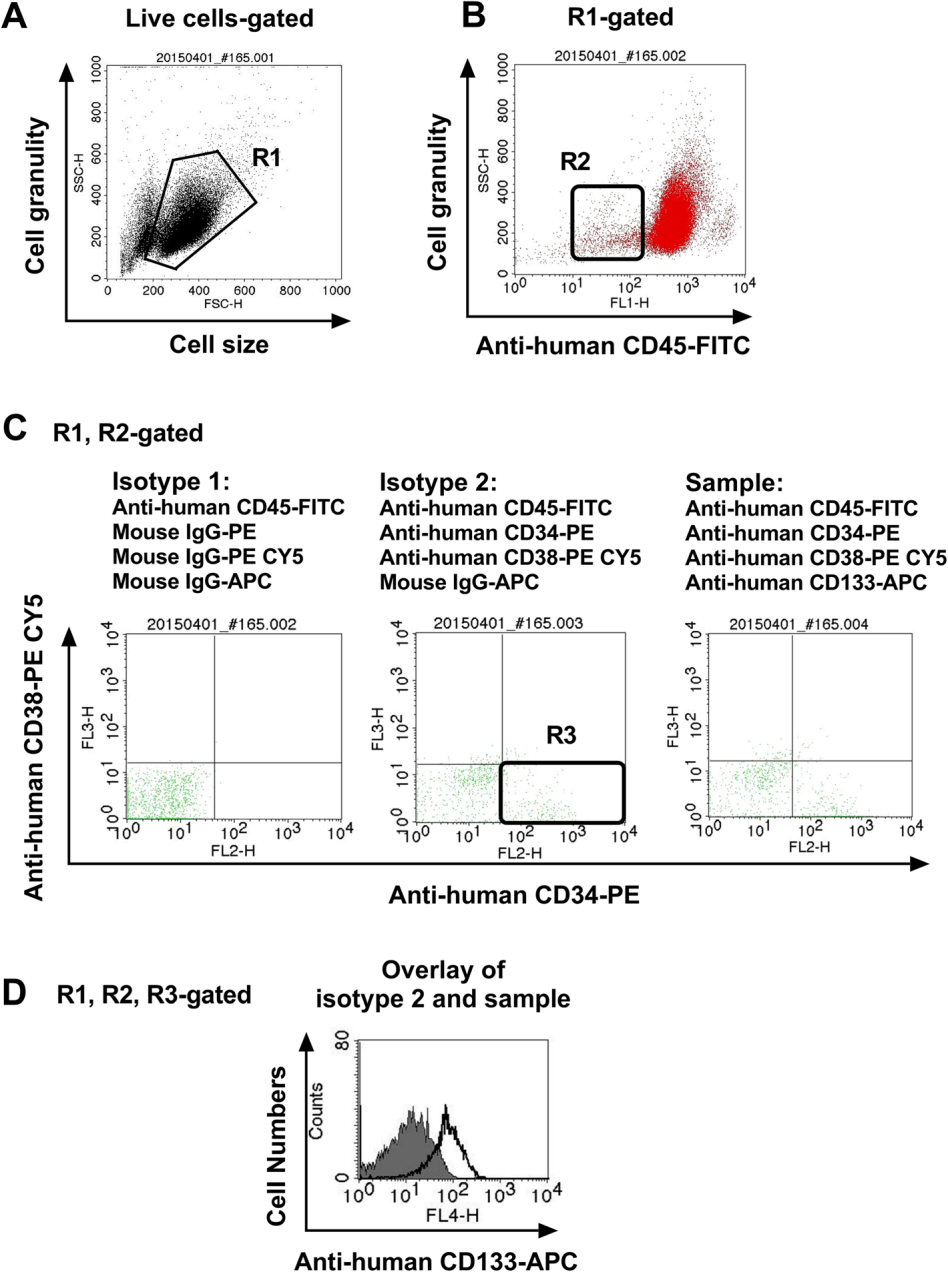
The process of four-color staining flow cytometry using monoclonal antibodies. The BMCs were collected and washed twice with FACS buffer. Cells were incubated with four antibodies against cell surface antigens, including CD45, CD34, CD38, and CD133 on ice for 30 min. **a, b** The live BMCs were collected, and SSC^low^ and CD45^dim^ cells were gated. **c, d** The BMCs were incubated with three types of combinations of monoclonal antibodies (mAbs) on ice for 30 min such as isotype control 1 (mouse anti-human CD45-FITC, mouse IgG-PE, mouse IgG-PE CY5 and mouse IgG-APC), isotype control 2 (mouse anti-human CD45-FITC, mouse anti-human CD34-PE, mouse anti-human CD38-PE CY5, and mouse IgG-APC), and sample (mouse anti-human CD45-FITC, mouse anti-human CD34-PE, mouse anti-human CD38-PE CY5, and mouse human CD133-APC). Cells were then washed twice with FACS buffer and analyzed using the FACSCalibur flow cytometer and CellQuest Pro software (BD Bioscience). Finally, the levels of CD45^dim^CD34^+^CD38^−^CD133^+^ cells, CD133 positive cells among the R1, R2, R3-gated cells were measured and the results were expressed as percentage change from the baseline conditions including isotype control 2. The filled histogram represents the isotype control 2, and the empty histogram represents CD45^dim^CD34^+^CD38^−^CD133^+^ cells

The BMCs were collected and washed twice with FACS buffer (PBS containing 0.3% BSA and 0.1% NaN_3_). The total bone marrow cell number used in the experiment was 4 × 10^6^ cells. Cells were incubated with four antibodies against each cell surface antigen, including CD45, CD34, CD38, and CD133 on ice for 30 min. First, live BMCs were collected, and SSC^low^ and CD45^dim^ cells were gated, as shown in Fig. 1a and b. And we always draw gates with the same criteria and select cells in the same section. The criteria are as follows: R1 Gate: live cells; R2 Gate: SSC-H, 100–500 and FL2-H, 10^1^–10^2^; R3 Gate: FL2-H, 10^2^–10^4^, FL3-H, 10^0^–10^1^. The BMCs were incubated with three combinations of monoclonal antibodies (mAbs) on ice for 30 min; these included isotype control 1 (mouse anti-human CD45-FITC, mouse IgG-PE, mouse IgG-PE CY5, and mouse IgG-APC), isotype control 2 (mouse anti-human CD45-FITC, mouse anti-human CD34-PE, mouse anti-human CD38-PE CY5, and mouse IgG-APC), and sample (mouse anti-human CD45-FITC, mouse anti-human CD34-PE, mouse anti-human CD38-PE CY5, and mouse human CD133-APC), as shown in Fig. 1c and Fig. 1d. Cells were then washed twice with FACS buffer and analyzed using the FACSCalibur flow cytometer and CellQuest Pro software (BD Bioscience) as shown Fig. 1. Finally, the counts of CD45^dim^CD34^+^CD38^−^CD133^+^ cells, CD133 positive cells among the R1, R2, R3-gated cells were measured, and the results were expressed as percentage change from the basal conditions including the isotype control 2. The 40,000 cells were used for flow cytometric acquisition in each sample tube.

### ELISA for cytokine measurement

Cell-free plasma from bone marrow samples of patients with AML was collected and frozen at − 80 °C. Plasma interleukin (IL)-1β, IL-6, IL-17, and IL-23 levels were measured using ELISA kits according to the manufacturer’s introductions (R&D Systems).

### Statistics

The data presented here represent the mean ± standard error of mean (SEM) of at least three independent experiments. All values were evaluated by one-way analysis of variance followed by Turkey range tests implemented in GraphPad Prism 7.0. Differences were considered significant at *P* < 0.05. For patients with AML, continuous variables were compared using the Student’s *t*-test, whereas categorical variables were analyzed using the Pearson chi-square test or Fisher’s exact test. Overall survival (OS) was calculated from the date of HCT to the date of death or last follow-up. Event-free survival (EFS) was defined from the date of HCT to the date of relapse or death from any cause. Survival probabilities were estimated by the Kaplan–Meier method. Univariate and multivariate analyses for OS, EFS, and relapse probability were performed using the log rank test and Cox proportional hazards model, respectively. The following variables were included in univariate analyses: CD45^dim^CD34^+^CD38^−^CD133^+^ cell proportion, age, white blood cell (WBC) count, platelet count, bone marrow blast percentage, cytogenetic risk groups, chemotherapeutic regimens, and immunophenotyping including CD7, CD33, CD34, and HLA-DR. Variables with a *P*-value < 0.1 in the univariate analyses were included in the multivariate analyses. The statistical analyses were performed with SPSS version 21.0 software (IBM Corp., Armonk, NY). For all analyses, the *P-*values were two-sided; a *P*-value of < 0.05 was considered statistically significant.

## Results

### CD45^dim^CD34^+^CD38^−^CD133^+^ cells are present in high numbers in the bone marrow of patients with acute myeloid leukemia

**Fig. 2:**
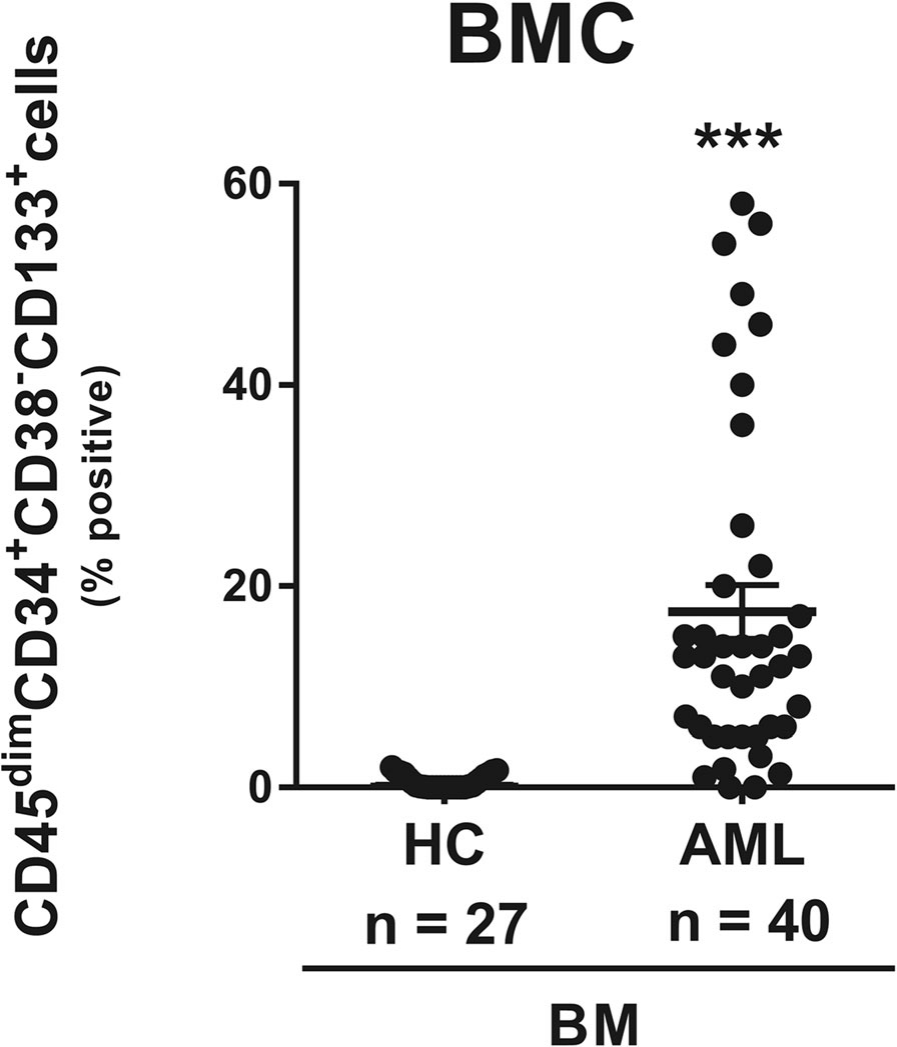
The CD45^dim^CD34^+^CD38^−^CD133^+^ cells are highly expressed in the bone marrow of patients with AML, but not in healthy controls. Bone marrow cells from healthy controls and AML patients were examined for the expression of the target antigens, CD45^dim^CD34^+^CD38^−^CD133^+^ cells. Data represent mean ± SEM representing three independent experiments from different AML patients. Significantly different from the control (*); ***, *P* < 0.001. HC, healthy controls; AML, acute myeloid leukemia patients

The work flow of the four-color flow cytometry experiments using monoclonal antibodies (mAbs) is shown in Fig. 1. As shown in Fig. 1a and b, live BMCs were collected and SSC^low^/CD45^dim^ cells were obtained. The BMCs were stained with various combinations of monoclonal antibodies for 30 min such as isotype 1, isotype 2, and sample (Fig. 1c). The CD133 positive cells in the R1, R2, R3-gated cells were measured using flow cytometry, and the results were expressed as percentage changes from the isotype 2 (Fig. 1d). A total of 40 AML patients were examined for the expression of the target antigens, CD45^dim^CD34^+^CD38^−^CD133^+^ on the surface of BMCs. These cells were present in high numbers in the bone marrow samples isolated from patients with AML, but not in those of healthy controls (Fig. 2). These results indicated that CD45^dim^CD34^+^CD38^−^CD133^+^ cells in bone marrow are potential AML stem cells.

### Elevated IL-1β, IL-6, IL-17 and IL-23 cytokine production of plasma in patients with AML

**Fig. 3:**
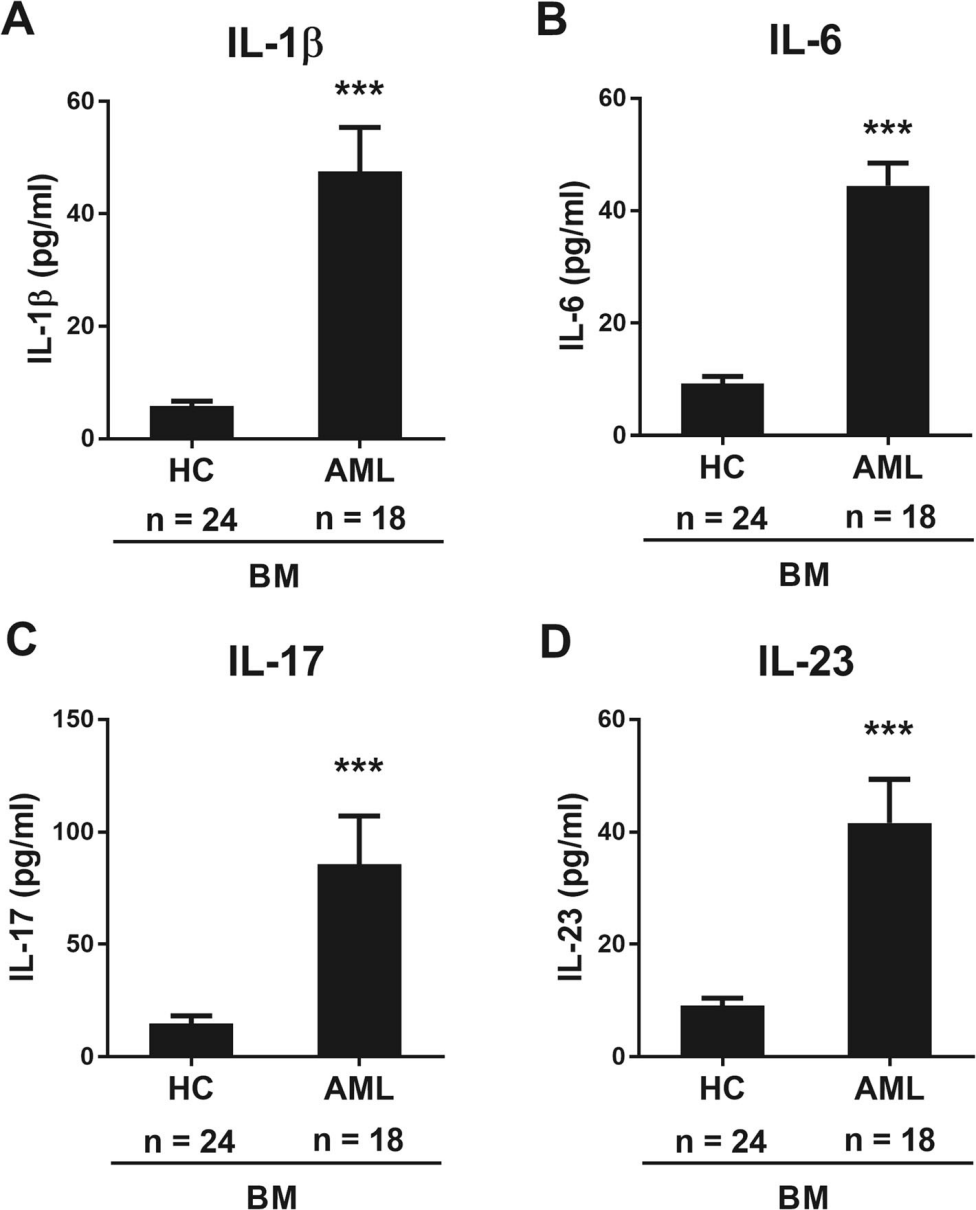
Quantification of the cytokines present in the plasma of healthy controls and AML patients. Cell-free plasma from bone marrow samples of AML patients was collected and frozen at − 80 °C. Plasma interleukin (IL)-1β, IL-6, IL-17, and IL-23 levels were measured using ELISA kits according to manufacturer’s introductions (R&D Systems). Data represent mean ± SEM from three independent experiments in different AML patients. Significantly different from the control (*); ***, *P* < 0.001. HC, healthy controls; AML, acute myeloid leukemia patients; BM, bone marrow

Recently, Th17 related cytokines such as IL-1β, IL-6, IL-17, IL-21, IL-22, and IL-23 play crucial roles in the pathogenesis of many diseases, including inflammatory diseases, autoimmune diseases, and cancers [24]. They have been shown related to Th17 cells. Especially, elevated frequencies of these cytokines in patients with AML have been associated with prognosis [25]. Therefore, we examined the levels of IL-1β, IL-6, IL-17 and IL-23 in the bone marrow plasma samples, which were matched to BMCs in AML patients. Plasma samples from the AML patients exhibited higher levels of IL-1β, IL-6, IL-17, and IL-23 than those from healthy controls (Fig. 3).

### The CD45^dim^CD34^+^CD38^−^CD133^+^ cells are prominently detected in the bone marrow of patients with AML and CML

**Fig. 4:**
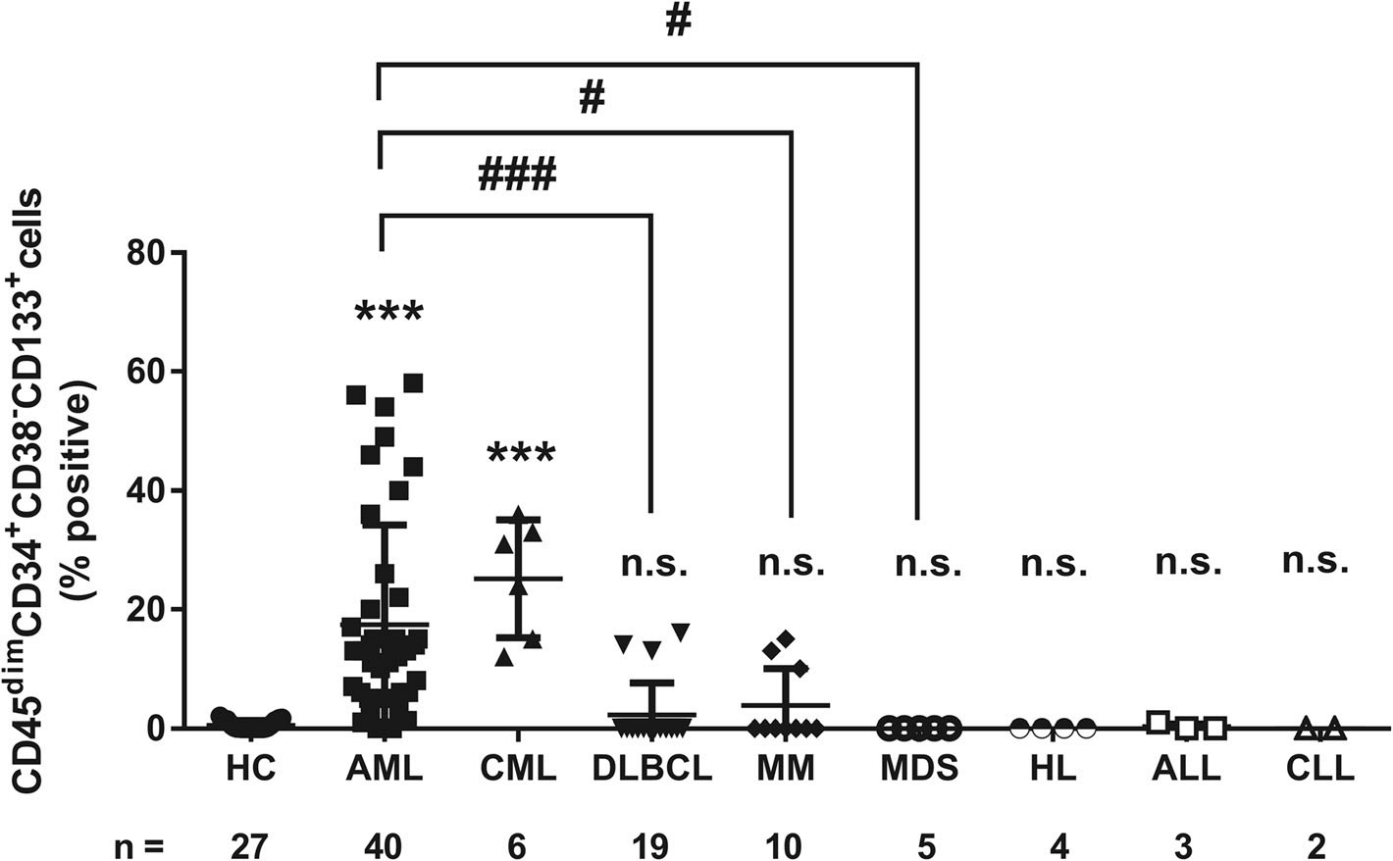
The CD45^dim^CD34^+^CD38^−^CD133^+^ cells are prominently detected in bone marrow of patients with AML and CML. As shown in Fig. 1, the CD45^dim^CD34^+^CD38^−^CD133^+^ cells were examined by four-color flow cytometry in diverse hematological malignancies including AML (*n* = 40), CML (*n* = 6), DLBCL (*n* = 19), MM (*n* = 10), MDS, (*n* = 5), HL (n = 4), ALL (*n* = 3), and CLL (*n* = 2). Data represent mean ± SEM from three independent experiments in different AML patients. Significantly different from the control (*) or AML (#); #: *P* < 0.05.; ***, ###: *P* < 0.001. HC, healthy controls; AML, acute myeloid leukemia; CML, chronic myeloid leukemia; DLBCL, diffuse large B-cell lymphoma; MM, multiple myeloma; MDS, myelodysplastic syndrome; HL, Hodgkin lymphoma; ALL, acute lymphocytic leukemia; CLL, chronic lymphocytic leukemia

As shown in Fig. 4, the CD45^dim^CD34^+^CD38^−^CD133^+^ cells were examined by four-color flow cytometry in diverse hematological malignancies including AML (*n* = 40), CML (*n* = 6), DLBCL (*n* = 19), MM (*n* = 10), MDS, (*n* = 5), HL (n = 4), ALL (*n* = 3), and CLL (*n* = 2). These cells are significantly detected in the bone marrow of patients with AML and CML, but not in those with DLBCL, MM, MDS, ALL, CLL, and HL. These results indicated that CD45^dim^CD34^+^CD38^−^CD133^+^ cells in bone marrow are potential of AML stem cells. In addition, these cells might be used for the detection of AML stem cells.

### Clinical characteristics according to levels of the CD45^dim^CD34^+^CD38^−^CD133^+^ cells

CD34^+^ AML and CD34^−^ AML among 36 AML patients evaluable for CD34 expression was noted in 30 patients and 6 patients, respectively. The proportion of CD45^dim^CD34^+^CD38^−^CD133^+^ cells in CD34- AML were significantly lower than CD34^+^ AML (median, 5.0% [range, 1–14%] vs. 13.5% [range, 1.8–58%], *P* = 0.001, respectively). And CD34^−^ AML showed tendency to have lower proportion of CD45^dim^CD34^+^CD38^−^CD133^+^ cells. FLT3-ITD mutation was rarely found in AML patients with higher counts of CD45^dim^CD34^+^CD38^−^CD133^+^ cells (≥10%) than in patients with fewer CD45^dim^CD34^+^CD38^−^CD133^+^ cells (< 10%) (0% vs. 23.1%, respectively*, P* = 0.031). In addition, higher counts of CD45^dim^CD34^+^CD38^−^CD133^+^ cells (≥ 20%) were significantly associated with lower levels of IL-17, as compared to lower CD45^dim^CD34^+^CD38^−^CD133^+^ cells (< 20%) (118.0 vs. 35.0 pg/ml, respectively, *P* = 0.028). However, there was no significant difference in IL-1β, L-6, and IL-23 levels based on the population of CD45^dim^CD34^+^CD38^−^CD133^+^ cells.

### High proportion of the CD45^dim^CD34^+^CD38^−^CD133^+^ cells predicts poor survival in AML patients

**Fig. 5:**
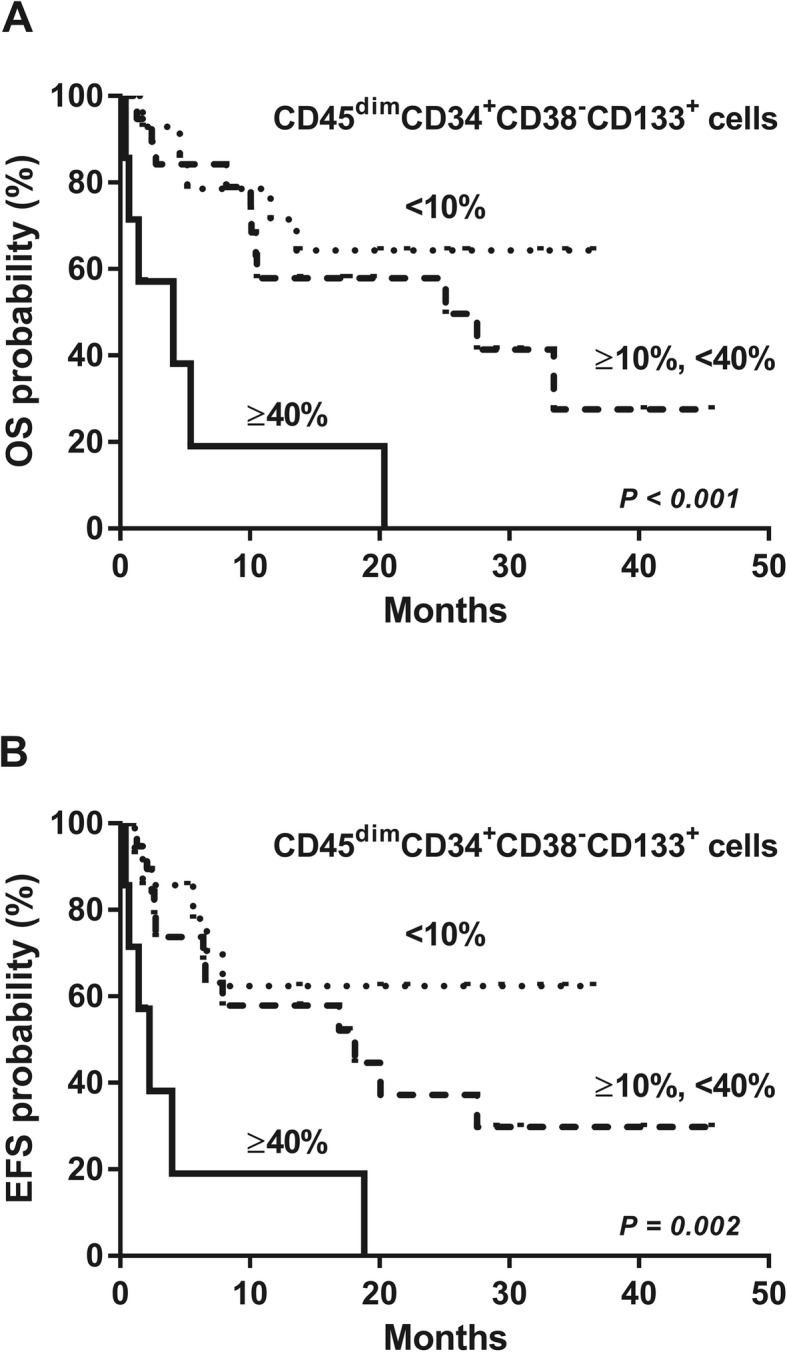
High proportion of the CD45^dim^CD34^+^CD38^−^CD133^+^ cells predicts poor survival in AML patients. **a** Higher CD45^dim^CD34^+^CD38^−^CD133^+^ cell proportion was significantly associated with worse OS (*P* < 0.001). **b** Poor EFS was significantly associated with higher proportion of CD45^dim^CD34^+^CD38^−^CD133^+^ cells (*P* = 0.002)

When we divided AML patients into three groups based on the percentage of CD45^dim^CD34^+^CD38^−^CD133^+^ cells (< 10%, 10–40%, and ≥ 40%), univariate analysis revealed that the 2-year OS rate was 64.3, 57.9, and 0%, respectively (*P* < 0.001) and the 2-year EFS was 62.3, 37.2, and 0% (*P* = 0.002), respectively (Supplementary Table 3). Among the three groups (CD45^dim^CD34^+^CD38^−^CD133^+^ cell proportions < 10%, 10–40%, and ≥ 40%), no significant differences were observed in baseline clinical factors including age (*P* = 0.085), white blood cell count (*P* = 0.397), platelet count (*P* = 0.737), and chemotherapy intensity (*P* = 0.158). Univariate analyses for OS and EFS in patients with AML revealed that older age (> 60 years) was significantly associated with worse OS than younger age (32.8% vs. 75% at 2-year, respectively, *P* = 0.041) (Supplementary Table 3). In addition, patients with higher marrow blast % (≥ 60%) showed significantly lower OS rates than those with lower marrow blast % (< 60%) (36.7% vs. 66.7%, *P* = 0.038) (Supplementary Table 3). Patients who were treated with intensive chemotherapy showed significantly better OS than those treated with hypomethylating agents (57.8% vs. 30.0%, *P* = 0.012). When we took into consideration other clinical parameters in univariate and multivariate analyses, higher percentage of CD45^dim^CD34^+^CD38^−^CD133^+^ cells (≥ 40%) was found to be an independent prognostic factor for OS (hazard ratio [HR], 6.052, *P* = 0.005) and EFS (HR, 9.028, *P* = 0.002) (Fig. 5. and Table 1). In addition, higher BM blast (%) ≥60% (HR, 2.607 < *P* = 0.049) and chemotherapy intensity (hypomethylating agents vs. intensive chemotherapy) (HR, 4.058, *P* = 0.010) were significant prognostic factors for OS in multivariate analysis.

**Table 1:** Multivariate analysis for patients with acute myeloid leukemia

	HR for OS	*P*-value	HR for EFS	*P*-value
**CD45**^**dim**^**CD34**^**+**^**CD38**^**−**^**CD133**^**+**^				
< 10% (*n* = 14)	1		1	
10- < 40% (n = 19)	1.859	0.276	2.731	0.089
> 40% (*n* = 7)	**6.052**	**0.005**	**9.028**	**0.002**
**BM blast (%)**				
< 60% (*n* = 16)	1		–	–
≥ 60% (*n* = 24)	2.607	0.049		
**Chemotherapy**				
Intensive chemotherapy (*n* = 30)	1		1	
Hypomethylating agent (*n* = 10)	4.058	**0.010**	4.829	**0.010**

*HR* hazard ratio, *OS* overall survival, *EFS* event free survival

## Discussion

The hypothesis that cancer stem cells including LSCs are responsible for the initiation, relapse, and drug resistance of cancers has caused a great deal of excitement in this area of research. The importance of cancer stem cells has been demonstrated in a variety of tumors [6, 25–29]. Especially, LSCs have unlimited capacity of self-renewal and are responsible for the maintenance of leukemia. Because selective eradication of LSCs could lead to considerable therapeutic benefits, there has been an interest in the identification and characterization of the LSC population that controls their development [30, 31]. Therefore, studies related to prognostically relevant and potentially reliable molecular targets are needed.

AML is a hematopoietic disease that is characterized by clonal growth and the accumulation of myelopoietic progenitor cells [31]. It is a devastating disease that is mostly incurable [4]. Moreover, the treatment for AML involves intense cytotoxic treatment as approximately 70% of the patients with AML are refractory to initial therapy or undergo relapse [2]. This is at least partially driven by the chemo-resistant nature of the LSCs that maintain the disease. Therefore, novel anti-LSC therapies could decrease the number of relapses and improve survival.

The first LSC compartment that was described had the CD34^+^CD38^−^ immunophenotype [1, 11]. The CD34^+^CD38^−^ compartment was shown to contain both CD34^+^CD38^−^ LSCs and normal hematopoietic stem cells (HSCs) [13]. Mawali et al. have proposed that CD34^+^CD38^−^CD123^+^ cells are AML LSCs [17]. In the present study, the CD45^dim^CD34^+^CD38^−^CD133^+^ cells were examined by four-color flow cytometry to define a more specific and prognostically significant LSC population (Fig. 1).

In the present study, CD34^+^ AML was found in 75% of patients with AML and 6 patients had CD34^−^ AML. The clinical implications of CD45^dim^CD34^+^CD38^−^CD133^+^ cells might be different in CD34^−^ AML. The CD34^−^AML had lower proportion of CD45^dim^CD34^+^CD38^−^CD133^+^ cells. Even though we could not evaluate prognostic impact of CD45^dim^CD34^+^CD38^−^CD133^+^ cells in CD34^−^ AML due to small number of patients, some portion of CD45^dim^CD34^+^CD38^−^CD133^+^ cells in CD34^−^ AML might contain normal hematopoietic stem cells as well as LSCs.

CD133 has been reported to be a cancer stem cell marker in solid tumors [14, 32–34]. Several studies have shown that CD133 positive cells have the capacity for self-renewal, differentiation, high proliferation, and forming tumors in xenografts [33, 34]. Although the precise function of CD133 remains unknown, it is associated with aggressive cancers and poor prognosis. CD133 is known to be required for tumor growth and survival [14, 29, 32]. However, in hematological malignancies including AML, the clinical implications of CD133 expression are not well known. Interestingly, CD45^dim^CD34^+^CD38^−^CD133^+^ cells are present in more numbers in the bone marrow of patients with AML, but not in healthy controls (Fig. 2). In a further study, only CD133 expression in AML need to be investigated if CD133 marker positivity regardless of CD34^+^CD38^−^ might be a significant marker for discriminating LSC and a prognostic biomarker. Moreover, the asynchronism of CD133^+^ expression should be also evaluated in CD34^−^ AML in the future. In other lymphoid hematologic malignancies such as lymphoma, MM, ALL, and CLL than AML, there could be some differences according to the percentages of both malignant cells and CD34^+^CD38^−^ compartments within bone marrows because of niche competition between two cell populations.

We also found increased production of IL-1β, IL-6, IL-17 and IL-23 in the bone marrow microenvironment of AML patients at the time of diagnosis (Fig. 3). These findings suggest that IL-1β, IL-6, IL-17 and IL-23 may be associated with leukemogenesis or pathophysiology of AML. Carey et al. also reported that IL-1 and IL-1β might be associated with AML cell growth [35]. IL-3 plays a key role within the network of cytokines involved in the regulation of hematopoiesis and leukemic blast formation. However, IL-3 has no prognostic significance [20]. As expected, the plasma samples from the AML patients at diagnosis exhibited higher levels of Th17 related cytokines, including IL-1β, IL-6, IL-17 and IL-23, than those from healthy controls (Fig. 3). To be honest with you, we expected these cytokines to have some degree of association with LSCs, but it was difficult to find the correlation in the experimental results. More specifically, the prognostic impact of IL-17 in AML is not clear, although higher serum IL-17 levels have been reported to be an adverse prognostic factor of AML in a univariate analysis of IL-17 by Han et al. [25]. In our results, however, IL-17 did not seem to have an adverse impact on prognosis of AML, because IL-17 was inversely correlated with the percentage of CD45^dim^CD34^+^CD38^−^CD133^+^ LSCs which was shown to be a significant negative prognostic marker, considering together clinical factors. There is little data regarding IL-23 levels in AML, although IL-23 levels have been reported to be associated with AML leukemogenesis and disease susceptibility in a previous study [36]. Based on our findings, it may be more advantageous to investigate T helper type 17 (Th17) cell or cell level associations than to monitor cytokines expressed in plasma to understand the association between LSC and Th17.

We applied the gate of CD45^dim^ population using the same criteria. Also, our results showed that the individual differences were large for CD45^dim^ population (Supplementary Fig. 1). In addition, the CD45^dim^CD34^+^CD38^−^CD133^+^ cells were prominently detected in the bone marrow of patients with AML and CML, but not in those with DLBCL, MM, MDS, ALL, CLL, and HL (Fig. 4). Moreover, the prognostic significance of LSCs has been reported in previous studies [1, 17]. Tervinjin et al. showed that higher CD34^+^CD45^−^LAP^+^ cell proportions were related to poor survival [1]. However, our study demonstrated that higher levels of the CD45^dim^CD34^+^CD38^−^CD133^+^ cells predict poor OS and EFS in AML (Fig. 5). These results also indicate that the CD45^dim^CD34^+^CD38^−^CD133^+^ cell compartment in the bone marrow could help discriminate between LSCs and normal hematopoietic stem cells, and can serve as a strong prognostic marker. Therefore, targeting CD45^dim^CD34^+^CD38^−^CD133^+^ cells could serve as a novel therapeutic strategy in AML. Future studies will focus on the elimination of the CD45^dim^CD34^+^CD38^−^CD133^+^ cells in patients with AML. Also, it needs to make sure that CD45^dim^CD34^+^CD38^−^CD133^+^ cells actually work as LSCs in the future. And it is necessary to assess whether the CD45^dim^CD34^+^CD38^−^CD133^+^ cells have the same characteristics as the stem cells. Therefore, our results indicate that CD45^dim^CD34^+^CD38^−^CD133^+^ cells have the potential of leukemic stem cells in acute myeloid leukemia.

## Conclusions

CD45^dim^CD34^+^CD38^−^CD133^+^ cells in AML might potentially serve as LSCs. Moreover, the high CD45^dim^CD34^+^CD38^−^CD133^+^ cell counts in AML patients served as a significantly poor risk factor for overall and event free survival. In addition, this cell population might represent a novel therapeutic target in AML.

## Supplementary information

**Additional file 1: Table S1.** Baseline characteristics of AML patients.

**Additional file 2: Table S2.** Patient characteristics.

**Additional file 3: Table S3.** Univariate analysis for AML patients.

**Additional file 4: Figure S1.** The expression of CD45^dim^ population on bone marrow cells in the study.

## Data Availability

All data generated or analyzed during this study are included in this published article and its additional files. Please contact the author Jae-Cheol Jo (jcjo97@hanmail.net) upon reasonable requests.

## Abbreviations

BM: Bone marrow
BMCs: Bone marrow cells
HC: Healthy controls
AML: Acute myeloid leukemia
CML: Chronic myeloid leukemia
DLBCL: Diffuse large B-cell lymphoma
MM: Multiple myeloma
MDS: Myelodysplastic syndrome
HL: Hodgkin lymphoma
ALL: Acute lymphocytic leukemia
CLL: Chronic lymphocytic leukemia
