# Supplementary material for: CD45dimCD34+CD38−CD133+ cells have the potential as leukemic stem cells in acute myeloid leukemia
Source: BMC Cancer. 2020 Apr 6;20:285. doi: 10.1186/s12885-020-06760-1 (PMC7137473; doi:10.1186/s12885-020-06760-1)
Supplement: Supplementary file 1 — Additional file 1: Table S1. Baseline characteristics of AML patients. [file 12885_2020_6760_MOESM1_ESM.docx]

**Supplementary Table 1. Baseline characteristics of AML patients.**

|  |  | CD45^dim^CD34^+^CD38^-^CD133^+^ cells, % | | |  |
| --- | --- | --- | --- | --- | --- |
| **Clinical parameters** | **No. (Total = 40)** | **< 10% (n=14)** | **10-<40% (n=19)** | **≥40% (n=7)** | ***P*-value** |
| Age, median (range), years | 61.5 (28-84) | 60.5 (31-82) | 59.0 (28-84) | 73 (62-76) | 0.085^b^ |
| M/F, no. | 20 (50%)/ 20 (50%) | 7 (50.0%) /7 (50.0%) | 9 (47.4%)/ 10 (52.6%) | 4 (57.1%)/ 3(42.9%) | 1.0^c^ |
| CD45^dim^CD34^+^CD38^-^CD133^+^ cells, %, median (range) | 13.0 (0-58) | 5.0 (0-8.0) | 14.0 (10.0-36.0) | 49.0 (40-58) | <0.001 |
| White blood cell, x 10^3^/mm, median (range) | 4.3 (0.6-268.8) | 3.7 (0.65-268.8) | 4.6 (0.64-128.3) | 5.9 (2.56-200.8) | 0.397^b^ |
| Hemoglobin, g/dL, median (range) | 8.4 (3.7-11.1) | 8.5 (5.1-11.1) | 7.5 (3.7-11.1) | 8.3 (6.6-10.9) | 0.536^b^ |
| Platelet, x 10^3^/mm, median (range) | 53 (19.0-353.0) | 77.5 (19.0-157.0) | 50.0 (19.0-182.0) | 49.0 (22.0-353.0) | 0.737^b^ |
| Peripheral blast, %, median (range) | 21.0 (0-94.0) | 8.0 (0-94) | 20.0 (0-87.0) | 58 (3.0-91.0) | 0.311^b^ |
| Bone marrow cellularity, %, median (range) | 79.5 (10-100) | 67.5 (10.0-95.0) | 83.0 (20.0-100) | 78.9 (41.3-99.0) | 0.540^b^ |
| Bone marrow blast, %, median (range) | 62.8 (8.5-93.5) | 54.8 (23.5-86.5) | 69.5 (8.5-93.0) | 70.0 (25.5-91.5) | 0.532^b^ |
| Immunophenotypic marker |  |  |  |  | 0.049 |
| CD34^d^ positive | 30 (75%) | 8 (26.5%) | 16 (53.3%) | 6 (20.0%) |  |
| CD34 negative | 6 (15%) | 4 (66.7%) | 2 (33.3%) | 0 (0%) |  |
| Cytogenetic risk classification^a^ |  |  |  |  |  |
| Favorable | 10 (25.0%) | 2 (14.3%) | 5 (26.3%) | 3 (42.9%) | 0.738^c^ |
| Intermediate | 20 (50.0%) | 8 (57.1%) | 9 (47.4%) | 2 (28.6%) |  |
| Poor | 10 (25.0%) | 4 (21.4%) | 5 (10.5%) | 2 (14.3%) |  |
| NPM1 mutation (+), no. (%) | 4 (10%) | 0 (0%) | 2 (10.5%) | 2 (28.6%) | 0.111^c^ |
| FLT3-ITD mutation (+), no. (%) | 3 (7.5%) | 3 (23.1%) | 0 (0%) | 0 (0%) | 0.065^c^ |
| FLT3 D835Y mutation (+), no. (%) | 1 (2.5%) | 0 (0%) | 1 (5.3%) | 0 (0%) | 1.0^c^ |
| CEBPA mutation (+), no. (%) | 1 (2.5%) | 1 (7.7%) | 0 (0%) | 0 (0%) | 0.526^c^ |
| Induction chemotherapy, no. (%) |  |  |  |  |  |
| Intensive chemotherapy (AD or AI 7+3) | 30 (75.0%) | 11(78.6%) | 16 (84.2%) | 3 (42.9%) | 0.130^c^ |
| Hypomethylating agents | 10 (25.0%) | 3 (21.4%) | 3 (15.8%) | 4 (57.1%) |  |

^a^According to NCCN 2017 prognostic risk classification.; AD, cytarabine+daunorubicin; AI, cytarabine+ idarubicin. ^b^ P-value was calculated by one way ANOVA test among three groups (CD45^dim^CD34^+^CD38^-^CD133^+^ cells <10%, 10-<40% and ≥40%). ^C^ P-value was calculated by Fisher’s exact test. ^d^CD34 positivity was defined as ≥20% expression of leukemic blasts.
