# Supplementary material for: CD45dimCD34+CD38−CD133+ cells have the potential as leukemic stem cells in acute myeloid leukemia
Source: BMC Cancer. 2020 Apr 6;20:285. doi: 10.1186/s12885-020-06760-1 (PMC7137473; doi:10.1186/s12885-020-06760-1)
Supplement: Supplementary file 2 — Additional file 2: Table S2. Patient characteristics. [file 12885_2020_6760_MOESM2_ESM.docx]

**Supplementary Table 2. Patient characteristics.**

| **Clinical parameters** | **No. (Total = 49)** |
| --- | --- |
| Gender |  |
| Male | 32 (65.3%) |
| Female | 17 (34.7%) |
| Median age (range; years) | 60 (23-80) |
| Pathology |  |
| Diffuse large B cell lymphoma | 19 (38.8%) |
| - Bone marrow involvement (+) | 3/19 (15.8%) |
| Multiple myeloma | 10 (20.5%) |
| - Median percentage of plasma cells on bone marrow | 17% (range, 10% - 45%) |
| Chronic myeloid leukemia | 6 (12.2%) |
| Myelodysplastic syndrome | 5 (10.2%) |
| Hodgkin lymphoma | 4 (8.1%) |
| - Bone marrow involvement (+) | 0/4 (0%) |
| Acute lymphoblastic leukemia | 3 (6.1%) |
| Chronic lymphocytic leukemia | 2 (4.1%) |
