# Supplementary material for: CD45dimCD34+CD38−CD133+ cells have the potential as leukemic stem cells in acute myeloid leukemia
Source: BMC Cancer. 2020 Apr 6;20:285. doi: 10.1186/s12885-020-06760-1 (PMC7137473; doi:10.1186/s12885-020-06760-1)
Supplement: Supplementary file 3 — Additional file 3: Table S3. Univariate analysis for AML patients. [file 12885_2020_6760_MOESM3_ESM.docx]

**Supplementary Table 3. Univariate analysis for AML patients.**

|  | **2y-OS** | **P-value** | **2y-EFS** | ***P*-value** |
| --- | --- | --- | --- | --- |
| **CD45^dim^CD34^+^CD38^-^CD133^+^** |  | **<0.001** |  | **0.002** |
| <10% | 64.3% |  | 62.3% |  |
| 10-<40% | 57.9% |  | 37.2% |  |
| >40% | 0% |  | 0% |  |
| **Age**, years |  | **0.041** |  | **0.077** |
| < 60 | 75% |  | 61.1% |  |
| > 60 | 32.8% |  | 24.7% |  |
| **WBC count,** /mm^3^ |  | 0.412 | 0.412 | 0.332 |
| < 40, x 10^3^ | 53.6% |  | 44.2% |  |
| ≥ 40, x 10^3^ | 40.0% |  | 30.0% |  |
| **Platelet count,** /mm^3^ |  | 0.128 | 0.412 | 0.332 |
| < 40, x 10^3^ | 37.5% |  | 44.2% |  |
| ≥ 40, x 10^3^ | 45.8% |  | 30.0% |  |
| **BM blast**, % |  | **0.038** |  | **0.084** |
| < 60 | 66.7% |  | 51.8% |  |
| ≥ 60 | 36.7% |  | 30.6% |  |
| **Chromosome^a^** |  | 0.734 |  | 0.703 |
| Favorable | 68.6% |  | 51.4% |  |
| Intermediate | 50.7% |  | 36.5% |  |
| Poor | 36.4% |  | 36.4% |  |
| **Chemotherapy** |  | **0.012** |  | 0.175 |
| Intensive chemotherapy | 57.8% |  | 43.3% |  |
| Hypomethylating agent | 30.0% |  | 40.0% |  |
| **CD 33** |  | **<0.001** |  | **0.001** |
| (+) | 42.0% |  | 42.0% |  |
| (-) | 0% |  | 0% |  |
| **CD 34** |  | 0.426 |  | 0.969 |
| (+) | 46.8% |  | 41.5% |  |
| (-) | 66.7% |  | 33.3% |  |
| **HLA-DR** |  | 0.386 |  | 0.323 |
| (+) | 54.5% |  | 44.3% |  |
| (-) | 42.9% |  | 28.6% |  |
| **CD 7** |  | 0.773 |  | 0.459 |
| (+) | 57.1% |  | 28.6% |  |
| (-) | 51.2% |  | 44.3% |  |

^a^According to 2017 NCCN guideline ; BM, bone marrow.
