# Supplementary material for: CD45dimCD34+CD38−CD133+ cells have the potential as leukemic stem cells in acute myeloid leukemia
Source: BMC Cancer. 2020 Apr 6;20:285. doi: 10.1186/s12885-020-06760-1 (PMC7137473; doi:10.1186/s12885-020-06760-1)
Supplement: Supplementary file 4 — Additional file 4: Figure S1. The expression of CD45dim population on bone marrow cells in the study. [file 12885_2020_6760_MOESM4_ESM.docx]

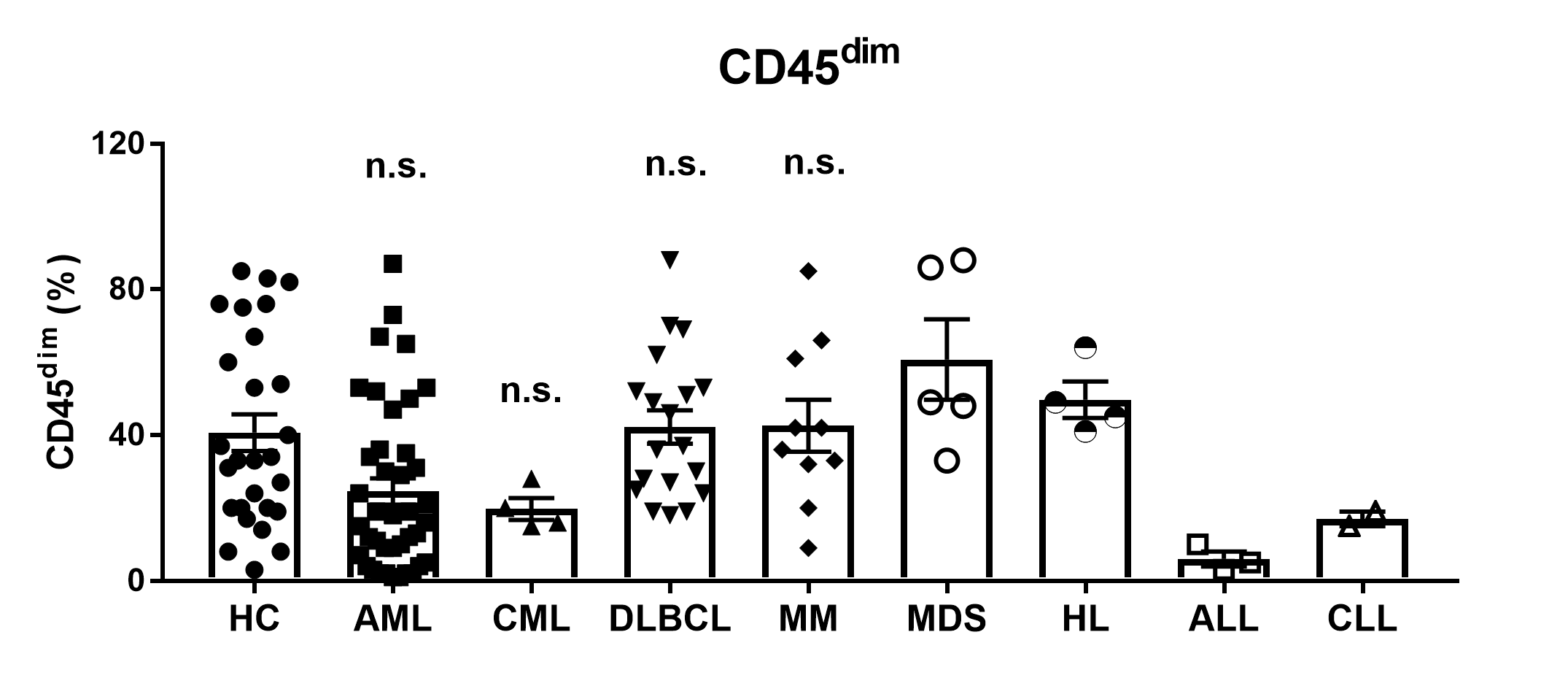


**Supplementary Figure 1**. The expression of CD45^dim^ population on bone marrow cells in the study. The CD45^dim^ cells were examined by flow cytometry in diverse hematological malignancies including AML (n = 40), CML (n = 6), DLBCL (n = 19), MM (n = 10), MDS, (n = 5), HL (n = 4), ALL (n = 3), and CLL (n = 2). Data represent mean ± SEM from three independent experiments in different AML patients. Significantly different from the control (*). HC, healthy controls; AML, acute myeloid leukemia; CML, chronic myeloid leukemia; DLBCL, diffuse large B-cell lymphoma; MM, multiple myeloma; MDS, myelodysplastic syndrome; HL, Hodgkin lymphoma; ALL, acute lymphocytic leukemia; CLL, chronic lymphocytic leukemia; n.s., no significance.
